# Measuring the Burden of Disease Due to Preterm Birth Complications in Korea Using Disability-Adjusted Life Years (DALY)

**DOI:** 10.3390/ijerph16030519

**Published:** 2019-02-12

**Authors:** Hyun Joo Kim, Min-Woo Jo, Seok-Hwan Bae, Seok-Jun Yoon, Jin Yong Lee

**Affiliations:** 1 Department of Nursing Science, Shinsung University, Dangjin 31801, Korea; hyjkim2012@gmail.com; 2 Department of Preventive Medicine, University of Ulsan College of Medicine, Seoul 05505, Korea; mdjominwoo@gmail.com; 3 Department of Radiological Science, Konyang University, Daejeon 35365, Korea; 4 Department of Preventive Medicine, Korea University, Seoul 02841, Korea; yoonsj02@korea.ac.kr; 5 Public Health Medical Service, Boramae Medical Center, Seoul National University College of Medicine, Seoul 07061, Korea; 6 Institute of Health Policy and Management, Medical Research Center, Seoul National University, Seoul 03080, Korea

**Keywords:** premature birth, disability-adjusted life year, burden of disease

## Abstract

The premature birth rate in Korea has increased from 13.5% in 2008 to 15.7% in 2013. The complications of premature birth are a major determinant of neonatal mortality and morbidities. The purpose of this study was to estimate the burden of premature birth in Korea using disability-adjusted life years (DALY). DALY consists of years of life lost (YLL) and years lost due to disability (YLD). In this study, preterm birth complications refer to nine diseases: P07, P22, P25, P26, P27, P28, P52, P77, P612, and H351 (International Classification of Diseases—10th Revision). YLL was calculated using mortality data from the 2012 National Health Insurance Data. YLD is a function of the prevalence, disability weight (DW), and duration of each complication. DW was determined by the Korean Disability Weight Study for National Burden of Disease in Korea 2013/2015. The burden of premature birth in Korea is 79,574 DALY (YLL: 43,725; YLD: 35,849). The burden for men (DALY: 43,603; YLL: 24,004; YLD: 19,599) is higher than that of women (DALY: 35,970; YLL: 19,720; YLD: 16,250). This study could provide essential data for evaluating the effects of policies intended to reduce preterm birth.

## 1. Introduction

Preterm birth is defined as giving birth earlier than 259 days since the last menstrual period or before 37 weeks of gestation [1]. There are multiple known causes of preterm birth, including multiple births, polyhydramnios, an incompetent cervix, anomaly of the uterus, hypertensive disorders of pregnancy, infection, and pregnancy-related complications [2]. Recently, increased marital age and the consequent increase in the number of high-risk pregnancies are becoming important factors in preterm birth. In the United States, the frequency of childbirth in women aged 35 years or older has increased from 5% in 1970 to 13% in 2000 [3]. The average age at first childbirth in Sweden rose from 25.9 years in 1970 to 28.9 years in 2000 [4]. Similarly, the frequency of childbirth in South Korean women aged 35 years or older has dramatically increased from 5% in 1996 to 14% in 2008 [5].

Preterm birth is an important cause of neonatal mortality as well as increased perinatal diseases due to the immaturity of various organs in the surviving newborns. Furthermore, preterm birth is a potential risk factor for permanent disabilities, such as impaired neurodevelopmental functioning and respiratory distress [6]. Despite socioeconomic and medical developments, preterm birth is increasing worldwide. In Japan, the preterm birth rate has increased from 4.1% in 1980 to 5.9% in 2010 [7], and in South Korea, the rate has increased from 4.5% in 2003 to 6.5% in 2013 [8]. One in every 10 newborns is born preterm worldwide, and more than 1 million babies die from preterm birth complications yearly [7]. Thirty-five percent of neonatal morbidity cases (3.1 million per year) are due to complications from preterm birth [9]; this is the second leading cause of death in infants aged 5 years or less, after pneumonia.

According to the 2010 Global Burden of Disease (GBD) study, preterm birth complications are responsible for 3.1% of the worldwide disease burden. However, owing to its low disease burden in South Korea (0.47%), there is little attention given to this issue. Nevertheless, one must be aware that preterm birth complications comprise the biggest proportion of neonatal diseases in South Korea [10]. Moreover, an increase in the frequency of preterm births will likely lead to increased preterm birth complications. Preterm birth complications are responsible for one-third of newborn deaths worldwide [9]. Moreover, preterm birth complications induce permanent disabilities, including cerebral palsy, learning disabilities, and vision and hearing disorders, putting a heavy burden on the family and society [11]. However, proper management during pregnancy can help to reduce the chances of preterm birth complications. Development of healthcare policies to reduce the likelihood of preterm birth complications is essential.

The World Health Organization has been measuring and comparing the world’s disease burden through the GBD study. However, an identical disease scope and disability burden were applied to all countries. Although this allowed for comparison of disease burden between countries, the GBD study results could not be used to set health service priorities and distribute limited healthcare resources, which are major advantages that studies on disease burden can offer. Our study took a different approach to overcome the limitations of the traditional GBD study and to measure the disease burden of preterm birth complications specifically in South Korea. In South Korea, the disease burden of preterm birth complications was measured using disability-adjusted life years (DALY) from the GBD 2000 study [12]. DALY is a single measure of disease burden due to disability or mortality determined from a national level of disease or disability, and it allows for comparison between countries. Using DALY helps not only in setting health service priorities but also in the distribution of limited healthcare resources. However, in the 2010 GBD study, all countries were given same disability weight in order to make comparisons between the countries [13,14]. Therefore, the disease burden of preterm birth complications calculated using the GBD study could not properly reflect the conditions in South Korea. Owing to the arguments surrounding the disability weight used in the GBD study, some studies have tried to estimate the disability weight to reflect the conditions of individual countries. In South Korea, some studies have attempted to estimate the disability weights of diseases, such as cancer and psychiatric disorders [15,16], and one study used an estimated disability weight to estimate the disease burden of a psychiatric disorder [17]. This study measured the disease burden of preterm birth complications using DALY.

## 2. Methods

### 2.1. Operational Definitions of Preterm Birth Complication

The causes of preterm birth complication were based on the hierarchical disease cause list from the GBD 2010 study [18]. The claims data of the National Health Insurance Service and Medical Aid Program in Korea were used. In this study, we operationally defined preterm birth complications using the International Classification of Diseases—10th Revision as referring to 10 diseases (Table 1): P07, P22, P25, P26, P27, P28, P52, P77, P612, and H351.

### 2.2. Patient Selection

In this study we calculated the DALY of preterm birth complication using data from Korea. We considered the appropriateness of defining the study population as under 5 years, similar to WHO. After conducting a literature review and consulting experts, we decided to expand the study population because there is limited data on this subject for Korea. Thus we extended the study to 0–9 years.

The criteria for the use of medical institutions were determined by the literature review and expert opinions. The patients selected for the study were aged 0–9 years who had visited at least 1 inpatient and 3 outpatient clinics for preterm birth complications.

### 2.3. Selection of the Standard for Severity Categorization 

We conducted literature searches and expert consultations to set the severity criteria. There was no literature indicating the severity of preterm birth complications. Therefore, we decided to follow the advice of experts. First of all, we analyzed the most frequent diseases of preterm birth complications through an analysis of healthcare screening assessment service claim data. As a result, the frequency of cerebral palsy (G80) was shown to be the highest. Analysis of the data from the Health Insurance Review and Assessment Service in 2012 showed that there were 2274 patients admitted for cerebral palsy (G80), which was 1.5 times the number of patients (1475) who were admitted for mental retardation (F70–79). Therefore, cerebral palsy (G80) was set as the standard for severity categorization of preterm birth complications [19,20,21]. Retinopathy of prematurity was categorized based on the ratio between visual impairment and severity. 

To assess the severity ratio between cerebral palsy and retinopathy of prematurity, data from the 2011 Survey of Disabled People were used [22]. The Survey of Disabled People is a national survey performed by the Ministry of Health and Welfare every 3 years, and the data include information on the category and severity of disabilities [22]. The severity rating in the survey is classified into six levels, from severe (level 1) to mild (level 6) [22]. In this study, we reclassified the severity of disability rating into mild, moderate, or severe. Level 1 was reclassified to the severe level, levels 2 and 3 were classified as intermediate, and levels 4–6 were considered mild. 

Cerebral palsy was categorized as a behavior disorder with or without cognitive impairment, similar to the categorization of the disability weight. From the analysis of the data on cerebral palsy from the Survey of Disabled People, the distribution of severity of disability in patients with behavior disorders without cognitive disorders was 2% mild, 90% moderate, and 8% severe, while the distribution in patients with cognitive disorders was 90% moderate and 10% severe. Retinopathy of prematurity was categorized using the same method, and the distribution of patients was as follows: 78% mild, 8% moderate, 14% severe, and 0.3% blinded. By applying the ratio calculated from the above numbers, years lost due to disability (YLD) values of behavior disorders with and without cognitive impairment and visual impairment and blindness from retinopathy of prematurity were calculated.

### 2.4. Disability Weight

Disability weight due to preterm birth complications was obtained using the Korean disability weight study [23]. The disability weight of preterm birth complications was calculated to reflect health status as follows [23]: epilepsy—treated, seizure-free or with recent seizures, untreated, or severe; distance vision—moderate, mild, or severe impairment, or vision blindness; motor impairment—mild, moderate, or severe; and motor plus cognitive impairments—mild or severe.

### 2.5. Calculation for DALY

To measure the burden of disease from preterm birth complications, we estimated the prevalence-based DALY that incorporates years of life lost (YLL) and YLD. The basic formula for DALY is shown in Equation (1):

DALY = YLL + YLD = N × L + P × DW,(1)

where N is the number of deaths, L is the standard life expectancy in years, P is the number of prevalent cases, and DW is the disability weight.

For YLL, the mortality rate was calculated using the cause-of-death data from Statistics Korea. In the case of causes that cannot or should not be considered an underlying cause of death, we followed the GBD’s redistribution algorithm for garbage codes. Years lost due to premature death were derived from the standard expected years of life lost. Life expectancy was based on the general population’s life expectancy by age, using the life tables of Statistics Korea in 2012.

The YLD was calculated using the number of prevalent cases and the disability weight. Disability weights were generated from a self-administered web-based survey of physicians and medical college students using a paired comparison for valuation method. The frame of the Korean Burden of Disease 2012 study and calculation of disability weights are described in more detail elsewhere [23]. 

The calculated YLD, YLL, and DALY values were standardized to the value per 100,000 members of the population. The population per 100,000 people was calculated based on the middle-aged population of Korean Statistical Information Service in 2012 [8]. The source of all data used for DALY calculation is shown in Table 2.

### 2.6. Sensitivity Analysis

A sensitivity analysis was performed because of the uncertainty of the data. The sensitivity analysis was performed by changing the prevalence and the number of deaths. First, only the prevalence was changed, second, the number of deaths was changed, and finally, the numbers of both were changed.

### 2.7. Ethics Statement

This study was approved by the Korea University Institutional Review Board (No. 1040548-KU-IRB-13-164-A-1[E-A-1]). Informed consent was waived by the board.

## 3. Results

### 3.1. YLD for Preterm Birth Complications

YLD values by severity are summarized in Table 3. The YLD value was 130.4 (males: 71.5; females: 58.9) for mild behavior disorders, 18,810 (males: 10,301.6; females: 8507.3) for moderate behavior disorders, and 1672 (males: 916; females: 757) for severe behavior disorders. The YLD value for moderate behavior disorders with cognitive impairment was 12,540 (males: 6867; females: 5673), with a higher YLD in men. The YLD value for severe behavior disorders with cognitive impairment was 1393 (males: 763; females: 630).

The YLD value was 782 (males: 408; females: 375) for mild retinopathy of prematurity, 80.0 (male: 42, female: 39) for moderate retinopathy, and 141 (males: 73; females: 67) for severe retinopathy. Similarly, the YLD value was higher in men. The YLD value for blindness was 301 and was higher in men (157) than in women (144).

The total YLD value from disabilities due to preterm birth complications was 35,849 (males: 19,599; females: 16,250) with men having higher YLD values. Based on age, patients less than 1 year of age had the highest YLD at 30,167 (males: 16,527; females: 13,639). 

### 3.2. YLL for Preterm Birth Complications

The YLL value due to premature death from preterm birth complications was 43,725 (males: 24,004; females: 19,720). Based on age, patients younger than 1 year of age had the highest YLL at 43,495.0 (males: 23,775; females: 19,720) (Table 4).

### 3.3. DALY Due to Preterm Birth Complications

The total DALY value from preterm birth complications was 79,574. The total YLD value was 35,849, and the YLL value was 43,725. YLL was 55% of total DALY, higher than YLD. The DALY per 100,000 population was 34,632 (YLD: 15,575 per 100,000 population, YLL: 19,057 per 100,000 population) (Table 5).

### 3.4. Sensitivity Analysis

**Figure 1:**
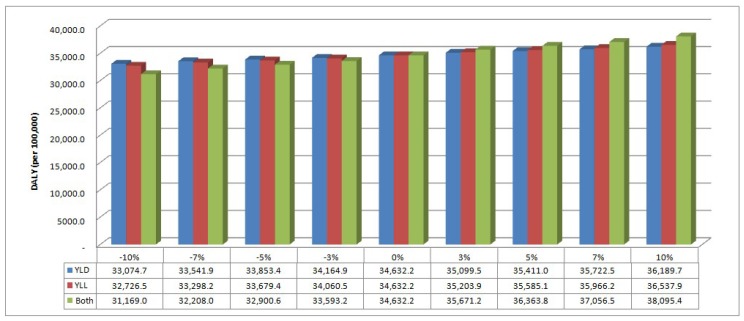
The results of sensitivity analysis. DALY: disability-adjusted life-years; YLD: years lost due to disability; YLL: years of life lost.

The sensitivity analysis was calculated assuming a decrease or increase of 3%, 5%, 7%, and 10%. As a result of performing the sensitivity analysis, it was shown that the change in YLL value is more influential on the DALY value than the YLD value is. The DALY value decreased further when the YLL value decreased by 10% (32,726.5 per 100,000 population). In addition, a 10% increase in the YLL value (36,537.9 per 100,000 population) showed a higher DALY value than a 10% increase in the YLD value (36,189.7 per 100,000 population) (Figure 1). However, the most effective decline in DALY value occurred when both the YLD value and the YLL value decreased.

## 4. Discussion

This study measured the burden of preterm birth complications using DALY. The disease burden of preterm birth complications was 79,574 (males: 43,603; females: 35,970). The total DALY of preterm birth complications was 34,632 per 100,000 population (YLD: 15,575 per 100,000 population, YLL: 19,056 per 100,000 population). The results of this study were lower than those of GBD 2010 (76,980 per 100,000 population) and GBD 2016 (62,031 per 100,000 population) worldwide [24,25]. 

The results of this study are different from previous studies for several reasons [24]. First, there was a difference in the definition of disease between the two studies. The GBD 2010 study included both the maternal and child-related burden of disease in the calculation of preterm birth complications. However, in this study, we excluded P010 (fetus and newborn affected by incompetent cervix) and P011 (fetus and newborn due to premature rupture of membranes) in order to calculate only preterm birth complications in terms of children, and we included retinopathy of prematurity (H351). Therefore, it is difficult to directly compare this study with GBD 2010 and 2016 research. In GBD 2010 and GBD 2016 in the Institute for Health Metrics and Evaluation (IHME), the burden of the preterm birth complication under 5 years in Korea was 47,699 and 33,833, respectively [25]. It should be noted that despite the exclusion of maternal-related disease, the burden of the preterm birth complication is higher in this study than in GBD 2016. These results suggest that the burden of disease caused by preterm birth complication in children has been underestimated. We also found that we needed to analyze the burden of maternal and child preterm birth complications separately, because children have a longer period of life to live with disabilities than individuals in motherhood. Therefore, the burden of disease of preterm birth complications should be estimated by dividing motherhood and child, and health policies should be different. 

The second difference in the methods of the two studies was the disability weights used. In this study, disability weights of preterm birth complication calculated from Korean data were used. The reason for using Korean disability weights is the issue of universality of disability weights being steadily raised since the early days of research on the burden of disease [26]. The advantage of applying universal disability weights is that it is possible to compare the degree of disease burden between countries. On the other hand, it does not reflect the social situation of each country. The GBD 2010 study therefore attempted to produce disability weights for more diseases in more countries. Nonetheless, there is still a limitation due to the number of countries that participated in GBD 2010’s disability weights output—only five countries (Bangladesh, Indonesia, Peru, Tanzania, and the USA) [24]. Therefore, in order to overcome these limitations, it is necessary to analyze using both the universal disability weights and the disability weights of each country. Especially, the increase in disease burden due to preterm birth complications is directly related to the increase of medical expenses and the decrease of productivity. Therefore, it is necessary to calculate the burden of diseases by using the disability weight of each country. 

Another reason for this study to be different from the GBD 2010 study is that it applied severity to the sequelae in calculating YLD. The disability weights were divided into mild, moderate to severe, and severe by disease sequelae, and the YLD were calculated. However, traditional GBD studies did not take severity into consideration [27]. It could be that the values calculated in the GBD 2010 study were underestimated, because this study did not consider the severity (mild: 0.299, moderate: 0.959, severe: 0.488). Therefore, the burden of preterm birth complications calculated in this study reflects the situation in Korea. It is indeed difficult to measure the disease burden after categorizing disease based on severity. Nevertheless, the reason for calculating the burden of disease by applying severity is that it can be an important basis for analyzing the effectiveness of these policies by setting medical policy priorities. The YLD of retinopathy of prematurity in this study was high as 1304 (mild: 782, moderate: 80, severe: 442). Unfortunately, a comparison with traditional GBD studies was impossible. The GBD study did not include retinopathy of prematurity (H351) [12]. However, retinopathy of prematurity is a disease that causes permanent disability if caused by premature birth [28]. Therefore retinopathy of prematurity is an important preterm birth complication and should be measured continuously. 

The YLD results indicate that disabilities caused by preterm birth complications almost always result in moderate or severe disabilities rather than mild disabilities. YLD values from disabilities due to preterm birth complications (including retinopathy of prematurity) were 912 for mild and 34,937 for moderate and severe complications, indicating a 40-fold higher disease burden. From these results, it appears that the majority of disabilities in the neonatal stage are greater than moderate-level severity. Therefore, a policy to minimize disabilities from preterm birth complications is urgently needed. However, healthcare policies regarding pregnancy and childbirth in South Korea include support for emergency childbirth, treatment for infertile couples, healthcare for the mother and the newborn, childbirth by women with disabilities, medical costs for preterm babies and babies with congenital abnormalities, congenital metabolic disorder examination and management, hearing disability examination, and full-body examination of children aged 6 years or less. The majority of these policies are supportive policies after childbirth; the only pre-birth management, which is the health management support system for the mother and the newborn, is very limited. The support is available only if the combined health insurance cost for the mother and her partner is below 65% of the national average monthly income and if the mother is within 40 days of her expected due date or 30 days after childbirth. Practically, there is no policy to prevent premature birth or to manage premature infants and preterm labor. Therefore, a policy to prevent and manage premature births is strongly required.

Compared to the GBD 2016 DALY of preterm birth complications with the Western-Pacific high-income countries group, Korea and Australia were similar but they were both higher than Japan and Singapore [29]. This difference was due to the YLL. The YLL of Korea was 1392, which was about 3 times higher than that of Japan (391 per 100,000 population) and Singapore (350 per 100,000 population) [29]. In this study, YLL was significantly high at 19,057 per 100,000 population. The difference between this study and the GBD 2016 study may be due to differences in the populations used. However, it is necessary to analyze the reason why preterm birth complication is high in future studies. In addition, it is necessary to analyze whether maternal or child causes of YLL increase. It is difficult to directly compare our study with others because of the differences in research methods, but the YLL for children in this study was 19,056 per 100,000 population. In order to analyze the reason why YLL is high in Korea, it is necessary to analyze the aspects of maternal and child health separately.

This study used national data, but there are uncertainties due to missing data when estimating the prevalence of preterm birth complications and deaths due to preterm birth. A sensitivity analysis was conducted to evaluate the uncertainty surrounding the DALY. The sensitivity analysis showed that DALY were lowered when YLL decreased more than when YLD decreased. However, when both YLD and YLL were decreased, DALY decreased significantly. On the other hand, DALY increased significantly when YLD and YLL were both increased. These results suggest that preterm birth management policy is important for continuous care for the prevention of death and disability.

Although the authors made an effort to ensure the validity of the methods by using all available data in South Korea, there were limitations. First, there was a lack of severity data to calculate the burden of preterm birth complications, and there was a limit to the calculation of years lost due to disability. To overcome these issues, the severity was calculated through a literature review and expert consultation. However, YLD may be underestimated or overestimated. Second, there is a possibility that underestimation was made because of the use of medical claim data. Nonetheless, this study is valuable in that it is the first study of the burden of preterm birth complications considering Korea’s disability weight and severity. 

## 5. Conclusions

The Korean government is making efforts to provide medical care for pregnant women and to provide vouchers for safe pregnancy and childbirth. Nonetheless, the results of this study and the GBD 2016 studies suggest that more effort is needed to reduce complications and death due to preterm birth. Therefore, a detailed analysis of complications of preterm delivery is needed. To this end, it is important to continuously analyze the burden of preterm birth complications among the mother and the child. It is also necessary to carry out this analysis in countries with high disease burden due to preterm birth complications. However, in this study, only the preterm birth complications of the child were analyzed. Therefore, it is necessary to study the maternal preterm birth complications.

## Figures and Tables

**Table 1:** Operational definitions of preterm birth complications.

ICD-10 Code	Causes
P07 (P070–P073)	Disorders related to short gestation and low birth weight, not elsewhere classified; excluded: low birth weight due to slow fetal growth and fetal malnutrition
P22 (P220–P229)	Respiratory distress of newborn; excluded: respiratory failure of newborn
P25 (P250–P258)	Interstitial emphysema and related conditions originating in the perinatal period
P26 (P260–P269)	Pulmonary hemorrhage originating in the perinatal period
P27 (P270–P279)	Chronic respiratory disease originating in the perinatal period
P28 (P280–P289)	Other respiratory conditions originating in the perinatal period; excluded: congenital malformations of the respiratory system
P52 (P520–P529)	Intracranial nontraumatic hemorrhage of fetus and newborn; included: intracranial hemorrhage due to anoxia or hypoxia; excluded: intracranial hemorrhage due to birth injury, intracranial hemorrhage due to maternal injury, intracranial hemorrhage due to other injury
P77	Necrotizing enterocolitis of fetus and newborn
P612	Anemia of prematurity
H351	Retinopathy of prematurity

Abbreviation: ICD-10, International Classification of Diseases—10th Revision.

**Table 2:** Source of Data.

Source of Data	
Mortality	2012 Korean Statistical Information Service deaths data
Prevalence	2012 National Health Insurance Service data
Severity	2011 Survey of Disabled People 2012 Disability Registration data 2012 Health Insurance Review and Assessment Service claim data
Life expectancy	2012 Korean Statistical Information Service, life tables
Disability weights	Korean Burden of Disease 2012 study and calculation of disability weights

**Table 3:** YLD for preterm birth complications.

	Severity	Age	Mild						Moderate						Severe					
Disease			Cause Prevalence			YLD			Cause Prevalence			YLD			Cause Prevalence			YLD		
			Male	Female	Total	Male	Female	Total	Male	Female	Total	Male	Female	Total	Male	Female	Total	Male	Female	Total
Behavior disorders ^a^		0	204	168	372	60.9	50.2	111.2	9171	7542	16,714	8795.0	7232.8	16,028.7	815	670	1486	781.6	642.5	1425.1
		1	23	20	43	6.9	6.0	12.9	1033	909	1942	990.6	871.7	1862.4	92	81	173	88.2	77.7	165.9
		2	6	5	11	1.8	1.5	3.3	280	214	494	268.5	205.2	473.7	25	19	44	24	18.2	42.2
		3	3	2	5	0.9	0.6	1.5	117	83	201	112.2	79.6	192.8	10	7	18	9.6	6.7	17.3
		4	1	1	2	0.3	0.3	0.6	55	41	96	52.7	39.3	92.1	5	4	9	4.8	3.8	8.6
		5	1	1	1	0.3	0.3	0.3	32	28	59	30.7	26.9	56.6	3	2	5	2.9	1.9	4.8
		6	0	1	1	0	0.3	0.3	22	23	45	21.1	22.1	43.2	2	2	4	1.9	1.9	3.8
		7	0	0	0	0	0	0	14	15	29	13.4	14.4	27.8	1	1	3	1.0	1.0	2.9
		8	0	0	0	0	0	0	11	10	21	10.5	9.6	20.1	1	1	2	1.0	1.0	1.9
		9	0	0	0	0	0	0	9	5	14	8.6	4.8	13.4	1	0	1	1.0	0	1.0
		Total	239	197	436	71.5	58.9	130.4	10,742	8871	19,614	10,301.6	8507.3	18,809.8	955	789	1743	915.8	756.7	1671.5
Behavior disorders with a cognitive impairment ^b^		0	-	-	-	-	-	-	6115	5028	11,142	5864.3	4821.9	10,685.2	679	559	1238	651.2	536.1	1187.2
		1	-	-	-	-	-	-	689	607	1295	660.8	582.1	1241.90	77	67	144	73.8	64.3	138.1
		2	-	-	-	-	-	-	186	143	329	178.4	137.1	315.5	21	16	37	20.1	15.3	35.5
		3	-	-	-	-	-	-	78	56	133	74.8	53.7	127.5	9	6	15	8.6	5.8	14.4
		4	-	-	-	-	-	-	37	27	64	35.5	25.9	61.4	4	3	7	3.8	2.9	6.7
		5	-	-	-	-	-	-	21	19	40	20.1	18.2	38.4	2	2	4	1.9	1.9	3.8
		6	-	-	-	-	-	-	14	15	30	13.4	14.4	28.8	2	2	3	1.9	1.9	2.9
		7	-	-	-	-	-	-	9	10	19	8.6	9.6	18.2	1	1	2	1.0	1.0	1.9
		8	-	-	-	-	-	-	7	6	14	6.7	5.8	13.4	1	1	2	1.0	1.0	1.9
		9	-	-	-	-	-	-	5	4	9	4.8	3.8	8.6	1	0	1	1.0	0	1.0
		Total	-	-	-	-	-	-	7161	5915	13,076	6867.4	5672.5	12,539.9	796	657	1453	763.4	630.1	1393.4
Retinopathy of prematurity ^c^		0	460	437	897	224.5	213.3	437.7	47	45	92	22.9	22	44.9	83	78	161	40.5	38.1	78.6
		1	238	204	441	116.1	99.6	215.2	24	21	45	11.7	10.2	22	43	37	79	21.0	18.1	38.6
		2	47	37	83	22.9	18.1	40.5	5	4	9	2.4	2.0	4.4	8	7	15	3.9	3.4	7.3
		3	26	23	48	12.7	11.2	23.4	3	2	5	1.5	1.0	2.4	5	4	9	2.4	2.0	4.4
		4	20	20	40	9.8	9.8	19.5	2	2	4	1.0	1.0	2.0	4	4	7	2.0	2.0	3.4
		5	21	16	37	10.2	7.8	18.1	2	2	4	1.0	1.0	2.0	4	3	7	2.0	1.5	3.4
		6	9	16	25	4.4	7.8	12.2	1	2	3	0.5	1.0	1.5	2	3	4	1.0	1.5	2.0
		7	8	6	14	3.9	2.9	6.8	1	1	1	0.5	0.5	1.0	1	1	3	0.5	0.5	1.5
		8	4	6	10	2	2.9	4.9	0	1	1	0	0.5	0.5	1	1	2	0.5	0.5	1.0
		9	3	4	7	1.5	2	3.4	0	0	1	0	0	0	1	1	1	0.5	0.5	0.5
		Total	835	768	1603	407.5	374.8	782.3	86	79	164	42.0	38.6	80.7	150	138	288	73.2	67.3	140.5
Blindness ^d^		0	-	-	-	-	-	-	-	-	-	-	-	-	177	168	345	86.4	82	168.4
		1	-	-	-	-	-	-	-	-	-	-	-	-	92	78	170	44.9	38.1	83.0
		2	-	-	-	-	-	-	-	-	-	-	-	-	18	14	32	8.8	6.8	15.6
		3	-	-	-	-	-	-	-	-	-	-	-	-	10	9	19	4.9	4.4	9.3
		4	-	-	-	-	-	-	-	-	-	-	-	-	8	8	15	3.9	3.9	7.3
		5	-	-	-	-	-	-	-	-	-	-	-	-	8	6	14	3.9	2.9	6.8
		6	-	-	-	-	-	-	-	-	-	-	-	-	3	6	10	1.5	2.9	4.9
		7	-	-	-	-	-	-	-	-	-	-	-	-	3	2	5	1.5	1.0	2.4
		8	-	-	-	-	-	-	-	-	-	-	-	-	2	2	4	1.0	1.0	2.0
		9	-	-	-	-	-	-	-	-	-	-	-	-	1	2	3	0.5	1.0	1.5
		Total	-	-	-	-	-	-	-	-	-	-	-	-	321	295	617	156.6	144.0	301.1

Abbreviation: YLD, years lost due to disability. ^a^ Disability weights of behavior disorders: mild—0.299, moderate and severe—0.959. ^b^ Disability weight of behavior disorders with cognitive impairment: moderate and severe—0.959. ^c^ Disability weight of retinopathy of prematurity: mild, moderate, and severe—0.488. ^d^ Disability weight of blindness: severe—0.488.

**Table 4:** YLL for preterm birth complications.

Age	Male			Female			Total YLL
	No. of Death	Life Expectancy	YLL	No. of Death	Life Expectancy	YLL	
0	305	77.95	23,774.5	233	84.64	19,720.4	43,495.0
1	2	77.19	154.4	0	83.86	0	154.4
2	0	76.22	0	0	82.89	0	0
3	1	75.24	75.2	0	81.90	0	75.2
4	0	74.25	0	0	80.91	0	0
5	0	73.27	0	0	79.92	0	0
6	0	72.28	0	0	78.93	0	0
7	0	71.28	0	0	77.94	0	0
8	0	70.29	0	0	76.95	0	0
9	0	69.30	0	0	75.95	0	0
Total	307		24,004.1	233		19,720.4	43,724.6

Abbreviation: YLL, years of life lost.

**Table 5:** DALY for preterm birth complications.

Age	YLD		YLL		DALY	
	Total	per 100,000	Total	per 100,000	Total	per 100,000
**0**	30,166.7	13,148.2	43,495.0	18,961.2	73,661.7	32,109.3
**1**	3779.8	1603.4	154.4	63.7	3934.2	1667.1
**2**	938.1	408.5	0	0.0	938.1	408.5
**3**	392.7	171.3	75.2	32.0	467.9	203.3
**4**	201.6	84.0	0	0.0	201.6	84.0
**5**	134.1	57.3	0	0.0	134.1	57.3
**6**	99.4	45.2	0	0.0	99.4	45.2
**7**	62.3	26.9	0	0.0	62.3	26.9
**8**	45.7	18.5	0	0.0	45.7	18.5
**9**	29.8	12.0	0	0.0	29.8	12.0
**Total**	35,849.0	15,575.4	43,724.6	19,056.9	79,573.6	34,632.2

Abbreviations: DALY, disability-adjusted life-years; YLD, years lost due to disability; YLL, years of life lost.

## List of Abbreviations

GBD	Global Burden of Disease
DALY	disability-adjusted life years
YLD	years lost due to disability
YLL	years of life lost
DW	disability weight
ICD-10	International Classification of Diseases-10th Revision
